# Novel long-acting brimonidine tartrate loaded-PCL/PVP nanofibers for versatile biomedical applications: fabrication, characterization and antimicrobial evaluation

**DOI:** 10.1039/d3ra02244g

**Published:** 2023-05-16

**Authors:** Samar A. Salim, Noha M. Badawi, Shahira H. EL-Moslamy, Elbadawy A. Kamoun, Baher A. Daihom

**Affiliations:** a Nanotechnology Research Center (NTRC), The British University in Egypt (BUE) Cairo 11837 Egypt elbadawy.kamoun@bue.edu.eg badawykamoun@yahoo.com; b Department of Pharmaceutics and Pharmaceutical Technology, Faculty of Pharmacy, The British University in Egypt (BUE) Cairo 11837 Egypt; c Bioprocess Development Department (BID), Genetic Engineering and Biotechnology Research Institute (GEBRI), City of Scientific Research and Technological Applications (SRTA-City) New Borg El-Arab City Alexandria 21934 Egypt; d Polymeric Materials Research Dep., Advanced Technology and New Materials Research Institute (ATNMRI), City of Scientific Research and Technological Applications (SRTA-City) Alexandria 21934 Egypt; e Biomaterials for Medical and Pharmaceutical Applications Research Group, Nanotechnology Research Center (NTRC), The British University in Egypt (BUE) Cairo 11837 Egypt; f Department of Pharmaceutics and Industrial Pharmacy, Cairo University Cairo Egypt; g Pharmaceutical Engineering and 3D Printing (PharmE3D) Lab, Division of Molecular Pharmaceutics and Drug Delivery, College of Pharmacy, University of Texas at Austin 78712 USA Baher.daihom@austin.utexas.edu Baher.daihom@pharma.cu.edu.eg

## Abstract

The global state of antibiotic resistance highlights the necessity for new drugs that can treat a wide range of microbial infections. Drug repurposing has several advantages, including lower costs and improved safety compared to developing a new compound. The aim of the current study is to evaluate the repurposed antimicrobial activity of Brimonidine tartrate (BT), a well-known antiglaucoma drug, and to potentiate its antimicrobial effect by using electrospun nanofibrous scaffolds. BT-loaded nanofibers were fabricated in different drug concentrations (1.5, 3, 6, and 9%) *via* the electrospinning technique using two biopolymers (PCL and PVP). Then, the prepared nanofibers were characterized by SEM, XRD, FTIR, swelling ratio, and *in vitro* drug release. Afterward, the antimicrobial activities of the prepared nanofibers were investigated *in vitro* using different methods against several human pathogens and compared to the free BT. The results showed that all nanofibers were prepared successfully with a smooth surface. The diameters of nanofibers were reduced after loading of BT compared to the unloaded ones. In addition, scaffolds showed controlled-drug release profiles that were maintained for more than 7 days. The *in vitro* antimicrobial assessments revealed good activities for all scaffolds against most of the investigated human pathogens, particularly the one prepared with 9% BT which showed superiority in the antimicrobial effect over other scaffolds. To conclude, our findings proved the capability of nanofibers in loading BT and improving its repurposed antimicrobial efficacy. Therefore, it could be a promising carrier for BT to be used in combating numerous human pathogens.

## Abstract 2

The global state of antibiotic resistance highlights the necessity for new drugs that can treat a wide range of microbial infections.

## Graphical abstract

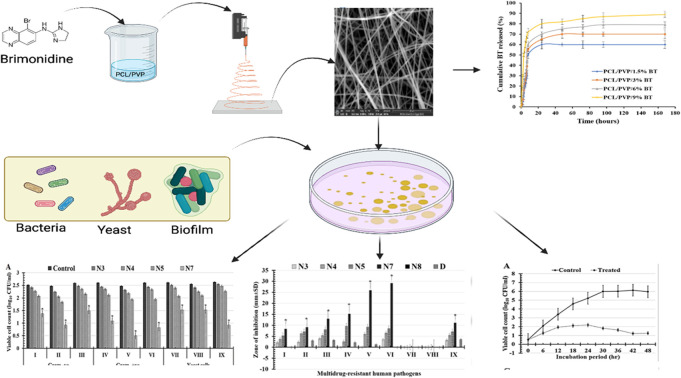

## 1 Introduction

Over the past few decades, multi-resistant pathogen-caused bacterial infections have become a serious global concern.^1^ The current state of antibiotic resistance on a global scale, as well as the pharmaceutical industry's reduced investment in antibiotic research, points to the need for specific cost-effective approaches to identify drugs for the action of many microbial infections.^2^ In addition, the process of developing new antimicrobials is extremely slow, and it is difficult to keep up with the evolution of bacterial resistance. As a result, there is a critical need for new medications, treatments, and alternative methods for battling multidrug-resistant bacterial pathogens, which are becoming an increasing clinical problem. Repurposing already-accepted medications, which is defined as using medications for conditions other than their primary indication and has a known pharmacology and toxicity, is an alternate method for hastening the development of new antimicrobials.^3^ The advantage of this trend to complete clinical data, which guarantees the safety profile of the repositioned drug on organs and cells. Furthermore, unlike traditional drug discovery, identifying new applications for existing medications is a proven shortcut that decreases the entire conditions and parameters associated with antibiotic development.

BT is a hydrophilic drug employed to treat glaucoma.^4^ Its pharmacological classification is an alpha 2 adrenergic agonist, which decreases aqueous humor production and increases uveoscleral outflow while promoting prostaglandin synthesis.^5^ It also has neuroprotective effects and reduces ischemia-induced optic nerve damage.^6,7^ It also has the benefit of working efficiently in normal-tension glaucoma,^8,9^ where the intraocular pressure (IOP) is less than 21 mm Hg but typical glaucomatous structural and functional optic nerve defects exist.^10^ It doesn't have any cardiovascular or pulmonary adverse effects that other glaucoma medications do such as Timolol^11^ or Betaxolol.^12^ New findings about the anti-microbial efficacy of antipsychotics^13^ cardiovascular^14^ anti-inflammatory,^15^ and anti-neoplastic drugs are reported in the literature.^16,17^ Nevertheless, only one alpha-2 adrenergic receptor agonist, where Guanabenz, was reported to have antiparasitic activity by selectively inhibiting dephosphorylation of the parasite eukaryotic initiation factor 2 alpha subunit, which is required for translation.^18^

The critical issues in drug delivery applications are poor drug stability and bioavailability, low drug loading, uncontrolled drug molecule release, and unsustainable drug delivery. Regarding this, electrospinning can alleviate some of these issues due to its unique characteristics, such as ease of use with tailored-made fiber properties and applicability to a variety of materials, including polymers with fiber sizes ranging from nanometers to micrometers.^19,20^ Additionally, electrospun fibers have gained recent attention due to their broad range of biomedical uses. Fibers with diameters in the nanometer range are called nanofibers, in which the specific surface area increases intensely. Polymeric nanofibers are suitable for many applications with high specific surface area requirements because of this inherent property. Natural polymers like chitosan, fibronectin, and gelatin,^21^ as well as synthetic polymers like poly lactic-*co*-glycolic acid (PLGA), polycaprolactone (PCL), and polyvinyl pyrrolidone (PVP) can be used for nanofibers synthesis or their various combinations.^22,23^ Among these synthetic polymers, PCL and PVP have both been employed extensively as biomaterials for medicinal applications. They are both biocompatible, biodegradable, and eco-friendly (non-hazardous) synthetic polymers. In comparison to other biodegradable polymers like PLGA, which exhibit very slow drug release, higher degradation times, and a highly acidic environment after degradation, PCL has the advantages of a moderate drug release rate, a lower absorbable time *in vivo*, and the creation of a minimal acidic environment during degradation.^24^ On the other hand, PVP is a water-soluble polymer with low toxicity and better adhesiveness that has been widely used to manufacture fibers using the electrospinning process due to its spinnability and fiber extraction.^22^

Lancina *et al.*, fabricated BT loaded dendrimer nanofibrous mats, and the results showed that the safety of the platforms *in vitro* and *in vivo* examinations but there was an accumulation of the BT directing to very fast of its release and out-control of its use daily.^25^

It's worth mentioning that no previous research on the antimicrobial activity potential of BT has been reported. Interestingly, no previous efforts for loading BT into nanofiber system was reported in literature for prolonging the release profile, and the current study is the first report in this regard. This study aims to formulate BT in different nanofiber formulations using combination of polymeric biomaterials based on PCL/PVP with different drug concentrations for the repurposed BT. The prepared BT nanofibers were explored for their potential antibacterial activity, as well as the spectrum of their activity against various clinical isolates of multidrug-resistant Gram-positive and Gram-negative pathogens.

## 2 Materials and methods

### 2.1. Materials

Polycaprolactone (PCL, Mwt 14 KDa) was purchased from Sigma-Aldrich, Japan, and polyvinyl pyrrolidone (PVP, Mwt 40 KDa) was bought from Thermo-Fisher, Germany. Dichloromethane (DCM purity ≥ 99%) and methanol (90%) were obtained from Fisher Scientific, UK. Orchidia Pharmaceutical Co. in Egypt kindly supplied Brimonidine tartrate (BT). The human pathogens that were employed in this work were *Escherichia coli*, *Pseudomonas aeruginosa*, *Klebsiella pneumonia*, *Staphylococcus aureus*, *Staphylococcus epidermidis*, *Bacillus cereus*, *Candida albicans*, *Candida tropicals*, and *Candida glabrata* were collected from Bioprocess Development Department, GEBRI, SRTA-City Alexandria, Egypt.

### 2.2. Preparation of BT-loaded PCL/PVP nanofibers

Different concentrations of BT (1.5, 3, 6, 9% w/v) were well dispersed into PCL/PVP solutions. After that, the solutions were electrospun to obtain PCL/PVP composite nanofibrous scaffolds. Typically, the different composition was set as follows; 5% PCL (w/v) prepared by dissolving exact weight into a mixture of DCM and methanol with a ratio (3 : 1) (w/v, *i.e.* 0.5 g/10 ml), and 30% PVP (w/v, *i.e.* 3 g/10 ml methanol). The ratio of blended polymers solution was fixed to be (1 : 1), and PCL : PVP total volume solution was 10 ml ready for electrospinning. PCL/PVP-BT solutions were kept under stirring at ambient conditions overnight to get a uniform and well-dispersed BT-loaded PCL/PVP blend solutions. The BT-loaded PCL/PVP solutions were electrospun (applied voltage of 23 kV, feed rate of 0.6 ml h^−1^) and collected on a width of 40 mm on a rectangle plate collector fixed at a distance of 15 cm from 22G needle. All electrospinning experiments were carried out at ambient conditions with a relative humidity of 45%.

### 2.3. Physicochemical properties of NFs

#### 2.3.1. Drug content

BT content in PCL/PVP NFs was determined by spectrophotometric method *via* absorbance at 248 nm using UV-spectrophotometer (Cary 5000 UV-vis-NIR spectrophotometer, Thermo-Fisher Scientific, USA). To calculate the amount of BT entrapped in each mat, fibrous scaffold discs (1 × 1 cm) were dissolved in a known volume of an organic solvent composed of DCM and methanol. Consequently, different concentrations were calculated from standard calibration curve of BT which is dissolved in the same organic solvent.^24^ The amount of BT and the productivity of drug loading is outlined as percent of drug content to the weight of the sample and as percent of calculated drug to the initial amount of drug added as shown in the following equations.

1 Drug Loading (DL) = amount of drug/amount of fibers × 100

2 Encapsulation Efficiency (EE) = amount of drug/initial amount of drug × 100

#### 2.3.2. Swelling ratio of BT loaded-PCL/PVP NFs

The swelling ratio of BT/NFs was determined by the concept of water uptake capacity. NFs with known weights (denoted as *M*_1_) were put in a weighing bottle containing 20 ml of PBS (pH 7.4) and incubated for 7 days at 37 °C. Afterward, NFs were placed on filter paper to remove the excess water on the surfaces and then weighted in wet condition (denoted as *M*_2_). The swelling ratio (%) of each sample was calculated by using the following equation.^26^3
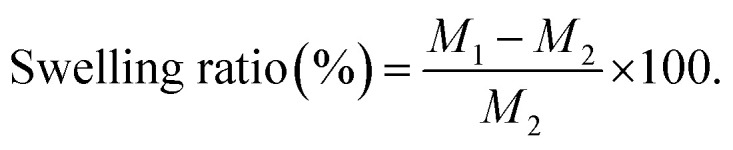


#### 2.3.3. *In vitro* release profile of BT loaded-PCL/PVP NFs

The *in vitro* release studies for 7 days were carried out in 20 ml of PBS (pH 7.4) using shaking water bath at 37 ± 0.2 °C at 50 rpm. At predetermined time intervals, 2 ml samples were withdrawn from each vial and replaced with an equal volume of the release medium. The concentrations of BT released were determined by spectrophotometric method at 248 nm, as above discussed.^24^

### 2.4. Instrumental characterization

#### 2.4.1. FTIR

FTIR analysis (Bruker Vertex 70, Germany), was used to establish the chemical binding among nanofiber components at the spectrum of 4000 to 400 cm^−1^ by revealing in a transmittance mode.

#### 2.4.2. XRD

X-ray spectra were collected using (Malvern Panalytical, Empyrean 3, UK), a Bruker D8 Advance using a tube with a copper anode at 40 keV and 30 mA. The diffraction patterns were obtained in the 2*θ* scan range of 5–70 with a step size of 0.05° and at the rate of 0.2 s per step.

#### 2.4.3. Scanning electron microscopy (SEM)

Scanning electron microscopy (FS SEM, Quattro S, Thermo-Scientific, USA) was performed to examine the surface morphology of nanofibrous scaffolds. The specimens were sputtered with a thin layer of Pt to reduce the surface charging of the samples. The SEM was operated using a backscattered electron detector at 5.0–7.0 kV and a working distance range of 7–13 mm to observe the surface features of the nanofibers. Image-Pro Plus (Media Cybernetics, Rockville, MD, U.S.A.) was used to quantitatively measure fiber diameter and distribution.^27^ The average diameter of different scaffolds was calculated using Image J software by detecting and analyzing of random 100 nanofibers.

### 2.5. Assessment of antimicrobial activity of BT and BT-loaded PCL/PVP NFs

In this work, the antimicrobial sensitivity of the evaluated formulations was investigated using several antimicrobial techniques, including the agar-well-diffusion procedure and the macro-dilution strategy.

#### 2.5.1. Evaluation of antibiotic susceptibility for examined human pathogens

In accordance with the CLSI (Clinical and Laboratory Standards Institute) procedure, disc-diffusion was used to test the investigated human pathogens for antibiotic susceptibility tests.^28^ One colony of each of the pathogens under investigation was picked at the log phase and seeded in tubes with 5 ml of sterile 0.85% saline solution. These tubes were then vortexed briefly. This was followed by an adjustment to McFarland standards of 0.5. Separately, 100 ml of the obtained cell suspensions were equally sprayed onto the surface of nutrient agar plates. Subsequently, these plates were allowed to dry before being topped with antibiotic discs. In these trials, ten antibiotic discs, including ciprofloxacin (CIP-5 mcg), oxacillin (OX-1 mcg), ampicillin (AP-10 g), fluconazole (FLC-25 mcg), clotrimazole (CC-10 mcg), ketoconazole (KT-30 mcg), aztreonam (ATM-30 g), and chloramphenicol (C-30 g), were investigated against each human pathogen individually. Finally, the zones of inhibition were detected after 24 hours of incubation at 37 °C, and these plates were classified as resistant (*R*, clear zone ≤5 mm) or sensitive (*S*, inhibitory zones >5 mm).

##### 2.5.1.1Preparation of the microbial pre-inoculum

In 10 ml of LB (Luria–Bertani) broth medium, which was prepared by dissolving 10 g of peptone and 5 g of yeast extract in 1000 ml of distilled water, a single colony from each pathogen was inoculated. These inoculums were then cultivated overnight at 37 °C and 200 rpm. The cell yield was separated out using centrifugation for 15 min at a speed of 10 000 rpm. To achieve a homogeneous cell suspension, the collected pellets were re-suspended in a 0.85% NaCl saline solution and gently mixed. The concentration of this microbial suspension was adjusted to 10^6^ CFU ml^−1^ using a saline solution.^28^ Two milliliters of each of these microbial suspensions were inoculated into 98 ml of the nutrient broth medium (0.5 g peptone, 1.5 g beef extract, 1.5 g yeast extract, 0.5 g sodium chloride, and 100 ml of distilled water). After inoculation, these flasks were placed in an incubator and kept at 37 °C for 24 hours.

##### 2.5.1.2 Well-diffusion assay

The tested formulations were labeled as (N3) for PCL/PVP/1.5% BT, (N4) for PCL/PVP/3.0% BT, (N5) for PCL/PVP/6.0% BT, (N7) for PCL/PVP/9.0% BT, (N8) for PCL/PVP and (D) for 1.5% BT. Briefly, the prepared nutrient agar plates were smeared with 100 μl of the freshly prepared microbial pre-inoculum using a sterile cotton swab. The next step was to drill wells through a sterile Cork borer with a 6 mm diameter, then add each tested formulation to its own well (each holding 50 μl). These plates were then kept at 37 °C for 24 h. After this incubation period, the diameter of inhibition zone that developed around the well was determined to evaluate antimicrobial efficacy of tested materials.^29,30^

##### 2.5.1.3 Anti-biofilm assay

This assay was applied in our study to evaluate the ability of each formulation to inhibit or prevent pathogenic microbial growth. A macro-dilution approach was used to evaluate the microbial turbidity in this experiment. In all these experiments, our formulations (N3, N4, N5, and N7) were examined against several human pathogens. In brief, the prepared pre-inoculum was diluted (10^6^ CFU ml^−1^) and cultivated using a shaker incubator at 37 °C and 180 rpm until it reached exponential growth (OD range: 0.2–0.3 at OD_600_). Then, 50 μl of each of the tested formulations were added individually. These treated tubes were aerobically incubated at 37 °C for 48 hours.^31–33^ All of the studied pathogens were tested against each formula in triplicate. Additionally, the control cultures were generated using diluted cultures that obtained no treatment. Firstly, with the aid of a spectrophotometer, the turbidity at OD_600_ was calculated using the average of six readings with the standard deviation (SD). Furthermore, using the controls' OD, the growth inhibition rate (% ± SD) was computed.

Secondly, the spread plate technique was applied to the anti-biofilm analysis. The colonies that formed biofilms were counted by swabbing 100 μl of each diluted culture onto a solid nutrient agar medium.^31^ Colony-forming units per milliliter (CFU ml^−1^) were used to calculate the results. To evaluate the level of biofilm inhibition, the logarithmic reduction of pathogen cells exposed to various formulations was determined. The computed percentage of biofilm inhibition was determined using counts of untreated control plates and treated plates to accurately predict the efficacy of the antimicrobial effects. The following formula, which used the CFU ml^−1^ readings of each control (*C*_untreated_) and its treated pathogen (*C*_treated_), was used to calculate the percentage of biofilm suppression:4
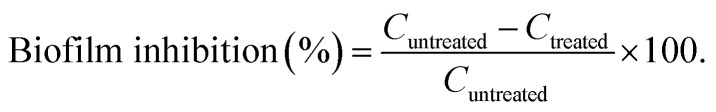


#### 2.5.2. Time-kill kinetics assay

In our experiments, we used pour-plates and macro-broth dilution methods to evaluate time-kill kinetics. A freshly prepared pre-inoculum of some of the pathogens was utilized to generate a pathogenic cell suspension containing 10^6^ CFU ml^−1^. These tubes were incubated at 37 °C with 200 rpm shaking for 2 hours to confirm that microbial growth was in the early logarithmic (exponential) phase. Then, 9 ml of fresh nutrient broth medium, 1 ml of tested formulations, and 1 ml of this exponentially growing microbial culture were mixed. These tubes were then incubated at 37 °C while being shaken at 200 rpm. Following that, the generated microbial growth was monitored at various periods of incubation (0, 6, 12, 18, 24, 30, 36, 42, and 48 h). The samples (100 μl) were diluted ten times in sterile saline (0.9% NaCl) under aseptic conditions. The final dilution was then swabbed at a volume of 100 μl onto nutrient agar plates. Colonies on each plate were counted after 24 hours of aerobic incubation at 37 °C, and the data was expressed as CFU ml^−1^. As a control, a culture setup without formulae was generated. Each experiment was carried out three times. For each time period, the percentage of logarithmic reductions for controls and pathogenic cells treated with different formulations was calculated. By charting the log_10_ CFU ml^−1^ of surviving pathogens with cultivation times, the time-kill curve for each pathogen was created.^34,35^

### 2.6. Statistical analysis

Experiments in this work were repeated three times. Statistical analysis was performed using the software Minitab® 18 statistical software. Statistical significance was considered at a *P* value <0.05.

## 3 Results and discussion

### 3.1. Fabrication of BT loaded-PCL/PVP NFs

Several spinning conditions were tested and optimized to achieve the accepted morphological nanofibers, in terms of smooth and bead-less nanofiber production. Herein, various concentrations of the BT (1.5, 3, 6, 9%, v/v) were embedded into the polymeric solution composed of PCL (5%) and PVP (30%) with a ratio (1 : 1) in 10 ml a total volume. These spinning solutions were loaded into a 10 ml plastic syringe connected to Teflon tube ended with 22G nozzle fixed into the clip spinneret. The high voltage affected each injected solution at different feed rates but all the solutions remained at the same distance from the collector almost 15 cm, as described in Table 1. All nanofiber scaffolds were collected on a conductive plate collector dressed with aluminum foil on a width 50 mm. The scaffolds were dried in a vacuum oven overnight to eliminate any residual solvent and then were stored till further investigation.

**Table 1:** Optimization of the spinning conditions of PCL/PVP & BT @ PCL/PVP scaffolds

Sample	Voltage (kV)	Feed rate (mL h^−1^)	Distance between tip & collector (cm)	Fiber morphology observation
PCL/PVP	20	0.6	15	Smooth, no beads formed and good NFs
PCL/PVP/1.5% BT	23	0.5	15	
PCL/PVP/3% BT	23	0.5	15	
PCL/PVP/6% BT	25	0.5	15	
PCL/PVP/9% BT	25	0.5	15	

### 3.2. Drug content

The mean loading of BT (DL, %) and encapsulation efficiency (EE, %) in different NFs scaffolds were calculated. All fabricated scaffolds showed high encapsulation efficiency of BT ranging from 97–99%, while the loading capacity of BT was average from 8 to 50%. These results recognized that the absence of drug loss during the electrospinning process and regular distribution of the drug through BT-loaded nanofibrous scaffolds. The high efficiency of loading BT may be due to using a polymeric composition of PCL and PVP with high concentrations of 5 and 30%; respectively, which allowed a high entrapment of BT in accordance with the reported findings of Amer *et al.*^36^

### 3.3. Instrumental characterization of BT loaded-PCL/PVP NFs

#### 3.3.1. FTIR analysis

**Fig. 1:**
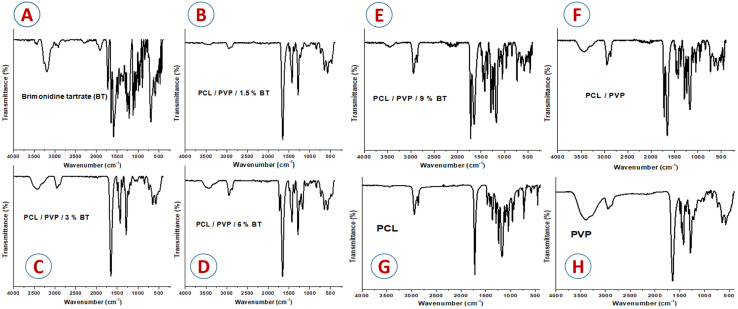
FTIR spectra of (A) Brimonidine tartrate (BT), (B) PCL/PVP/1.5% BT, (C) PCL/PVP/3% BT, (D) PCL/PVP/6% BT, (E) PCL/PVP/9% BT, (F) PCL/PVP, (G) PCL, and (H) PVP.

The spectrum of PCL (Fig. 1G), shows the absorbance peaks of C=O and C–O at *ν* 1724 and 1174 cm^−1^, respectively. On the other hand, PVP absorption spectrum in (Fig. 1H) revealed the vibration peaks of C=O and C–N at *ν* 1664 and 1441 cm^−1^, respectively^37,38^ A broad peak was detected at *ν* 3408 cm^−1^ which is associated with O–H group. The index peaks of PCL and PVP were observed in the blending PCL/PVP scaffold as shown in Fig. 1F. Moreover, no change was perceived in the peak at *ν* 2950 cm^−1^ that is related to the alkanes group of the polymer backbone.^37^ In Fig. 1A, the characteristics vibration peaks of BT were observed at *ν* 1482, 1574, 1648, 3200 cm^−1^ related to stretching C–C, N–H;, N=C, and NH stretching; respectively.^39^ As showed in the Fig. 1D and E, the characteristics vibration peaks located at *ν* 1729 and 1180 cm^−1^ were observed in PCL/PVP NFs loaded with high concentration of BT (6 and 9%, v/v) which related to tartrate (stretching vibrations of the C=O in carboxylate group).^40^ The scaffolds loaded with (1.5 and 3%, v/v) BT showed weak peak at *ν* 1185 cm^−1^ and this can be explained as the low concentration of drug and capsulation of drug into fibers as represented in Fig. 1B and C. These observations thus designate that BT was successfully embedded into PCL/PVP NFs.

#### 3.3.2. XRD analysis

**Fig. 2:**
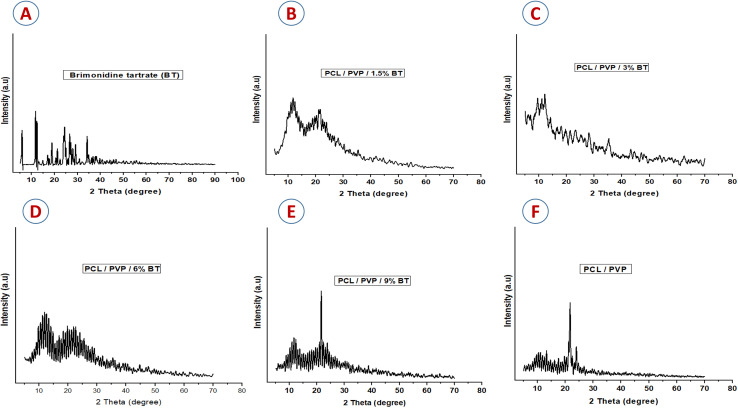
XRD diffractometry patterns of (A) Brimonidine tartrate (BT), (B) PCL/PVP/1.5% BT, (C) PCL/PVP/3% BT, (D) PCL/PVP/6% BT, (E) PCL/PVP/9% BT, and (F) PCL/PVP.

The crystallographic structure of plain and drug-loaded nanofibers scaffolds was investigated by XRD patterns (Fig. 2). The diffraction patterns show that the embedded drug made changes in the crystallinity of NFs^24^ The diffraction scan of BT powder presented various sharp intensity bands at diffraction angles (2*θ*) of 11.9°, 16.9°, 18.7°, 21.3°, 24.1°, 26.6°, 29.3°, 34.1°, and 34.6°, indicated the crystallinity structure of BT.^41^ As shown in Fig. 2F, two strong bands at 2*θ* = 21.5 and 23.8° were recorded accredited to the diffraction of the (110) lattice plane and the (200) lattice plane of semi-crystalline PCL; respectively. The electrospinning method decreased the crystallinity of PVP so displaying no bands. Moreover, blending PCL with PVP was exhibited to mask the crystalline structure of PCL, this observation is completely coupled with Nagiah *et al.*^42^ All loaded NFs scaffolds with different concentrations of BT showed halo diffractogram patterns that confirm the complete conversion of BT to an amorphous arrangement and its uniform embedding into PCL/PVP scaffolds as represented in Fig. 2B–E. It is proved that, the absence of intense sharp bands attributed to crystalline drug molecules in the diffractogram of the loaded scaffold validates that the drug which is present in an amorphous state in PCL/PVP NFs.^41,43^

#### 3.3.3. SEM investigation

**Fig. 3:**
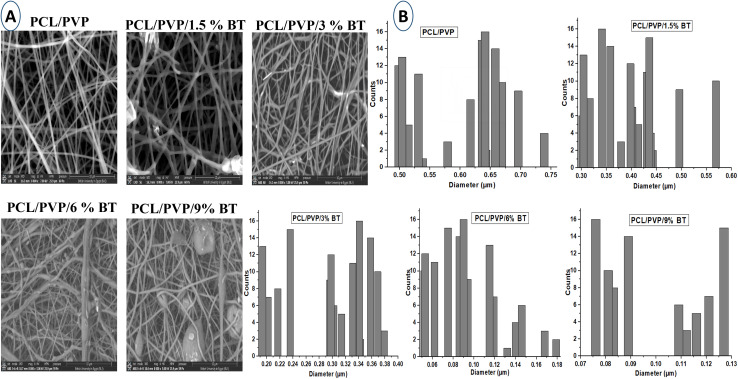
(A) SEM images of PCL/PVP, PCL/PVP/1.5% BT, PCL/PVP/3% BT, PCL/PVP/6% BT, PCL/PVP/9% BT, (original magnification 8000×; scale 10 μm), and (B) the average diameter of nanofibers for loaded and unloaded scaffolds.

SEM images of the PCL/PVP scaffolds with different concentrations of BT (1.5, 3, 6, and 9%, v/v), are shown in Fig. 3A. Scaffolds with high concentrations of BT (6 and 9% BT) revealed the encapsulation of the drug into the fibers in a spindle form due to the hydrophilicity of the drug. Fig. 3B shows the average diameter of the NFs. The mean diameter of plain nanofiber was 650 nm, which was then reduced to (450, 360, 100, 80 nm) by the addition of (1.5, 3, 6, and 9%, v/v of BT); respectively. This could be attributed to the pulling-down effect of the threads towards the collecting plate.^44^ Moreover, the diameter reduction of the loaded nanofibers could be due to the improvement of the electrical conductivity. These results agree with Afrashi *et al.*, who observed that the diameter of PCL/PVP NFs decreased by adding the antifungal drugs (fluconazole and ketoconazole), where the diameter of the plain scaffold was 656 nm then dropped to 439 and 416 nm by adding 15% of fluconazole and ketoconazole, respectively.^38^ Moreover, Zamani *et al.* recorded that, increasing metronidazole benzoate dose showed an enhancement in the polymeric solution conductivity, which led to a reduction of viscosity, and hence in the diameter of nanofibers.^45^

### 3.4. Physicochemical measurements of BT loaded PCL/PVP NFs

#### 3.4.1. Swelling ratio (%)

**Fig. 4:**
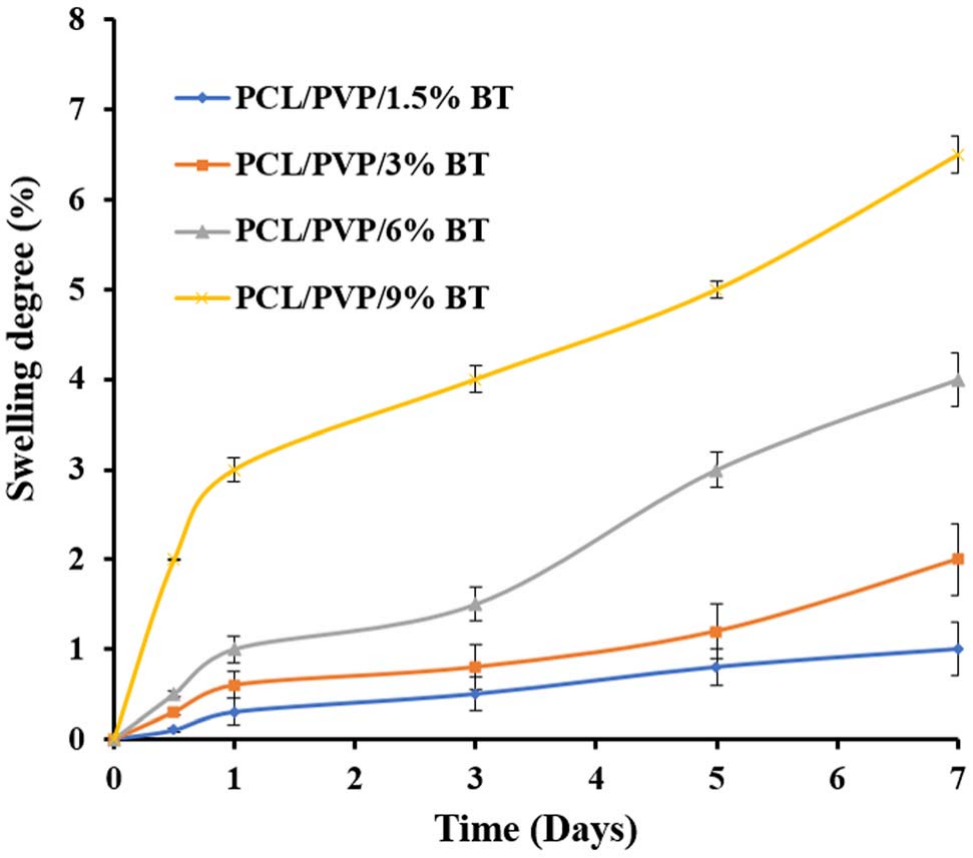
Swelling ratio of different BT-loaded nanofibrous scaffolds.

The swelling ratio has a significant effect on the released drug from nanofibers.^46^Fig. 4 shows the degree of swelling of BT-loaded PCL/PVP nanofibers at room temperature for 7 days. The degree of swelling reached 1% (PCL/PVP/1.5% BT), 2% (PCL/PVP/3% BT), 4% (PCL/PVP/6% BT), and 6.5% (PCL/PVP/9% BT), after 7 days. It was noticed that, as the proportion of BT in PCL/PVP nanofibers increased, the degree of swelling also increased. This phenomenon could be attributed to the hydrophilicity of BT which resulted in a higher degradation rate of the PCL/PVP nanofibers and thus, released BT enhanced, as well.^47^

Therefore, NFs scaffolds exhibit moderate weight loss% manners in PBS; where (PCL/PVP/9% BT) demonstrate the maximum degree of degradation; because it records the greatest swelling ratio, compared to scaffold loaded with the lowest concentration of BT (PCL/PVP/1.5% BT). This can be attributed to the amorphous structure of different loaded scaffolds as abovementioned were proven by XRD investigation, which thus permit water to diffuse through pores and expand the scaffolds even further.

#### 3.4.2. *In vitro* release study

**Fig. 5:**
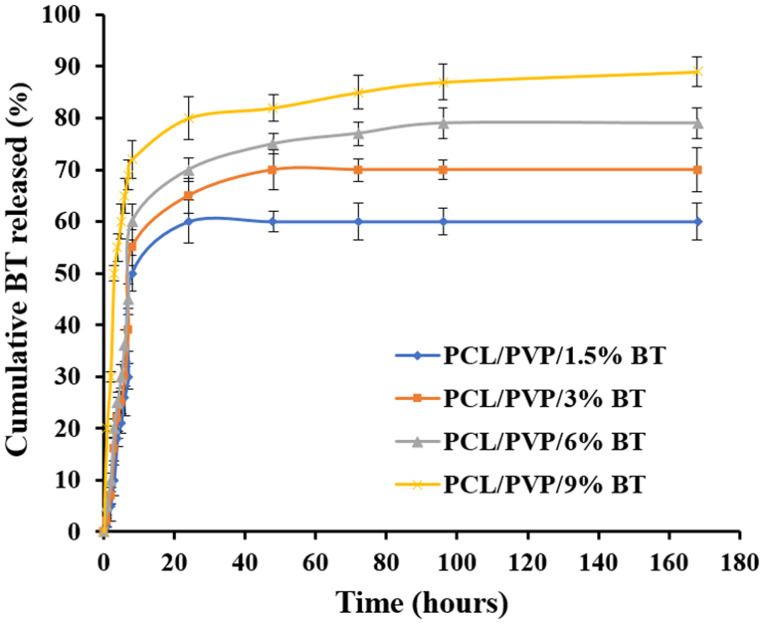
*In vitro* BT release profiles from different nanofibers.

The drug release profiles of the BT-loaded PCL/PVP nanofibrous scaffolds are shown in Fig. 5. It was observed that BT was released from the nanofibers by 21% (PCL/PVP/1.5% BT), 25% (PCL/PVP/3% BT), 30% (PCL/PVP/6% BT), and 60% (PCL/PVP/9% BT) during the first 5 h, followed by a sustained release during the subsequent 7 days. It was noticed that, as the BT concentration increased, the drug release was enhanced which might be owing to the accumulated drug molecules on the NFs surfaces, leading to a higher initial burst release.^48^ Also, it could be due to that the release profile depends mostly on polymer degradation and drug diffusion.^49^ It is worth mentioning that swelling ratio results were in good harmony with the drug release outcomes. As displayed, the PCL/PVP nanofiber scaffolds demonstrated typical biphasic release profiles and this result is in agreement with Yu *et al.* who reported a biphasic release behavior of vancomycin hydrochloride from PVP/PCL core/sheath fiber mat.^50^

### 3.5. Antimicrobial activity of BT and BT-loaded PCL/PVP NFs

The antimicrobial sensitivity of the formulations under evaluation was examined using a variety of antimicrobial techniques, such as the diffusion strategy (agar-well-diffusion test) to survey the antimicrobial activities (clear zones). Furthermore, the dilution strategy (macro-dilution tests) was employed to examine the damaged microbial biofilms, as well as the time-kill curve was determined by counting viable colonies.

#### 3.5.1. Antibiotics susceptibility analysis

The results of evaluating the studied pathogens' susceptibility to antibiotics are shown in Table 2. A startling prevalence of antibiotic resistance to seven different types was discovered when the pathogens *Klebsiella pneumoniae*, *Bacillus cereus*, and *Candida tropicalis* were tested for antibiotic susceptibility. Five of the tested antibiotics were subsequently found to be ineffective against *Escherichia coli*, *Candida albicans*, and *Staphylococcus aureus*. Four of the studied antibiotics were ineffective against *Staphylococcus epidermidis*, compared to only three were recorded against *Candida glabrata* and *Pseudomonas aeruginosa*. Finally, most pathogens possess a significant resistant to at least three altered types of antibiotics. According to previous reports, multidrug-resistant human pathogens were defined as human pathogens that were resistant to three or more types of antibiotic.^51–53^ Therefore, all of the pathogens we investigated were classified as multidrug-resistant human pathogens.

**Table 2:** Results of an antibiotic susceptibility test for human pathogens employing ciprofloxacin (CIP-5 mcg), oxacillin (OX-1 mcg), ampicillin (AP-10 g), fluconazole (FLC-25 mcg), clotrimazole (CC-10 mcg), ketoconazole (KT-30 mcg), aztreonam (ATM-30 g), and chloramphenicol (C-30 g)^a^

Human pathogens	Antibiotic sensitivity discs									
	FLC-25 mcg	C-30 μg	E-15 μg	ATM-30 μg	AP-10 μg	CIP-5 mcg	KT-30 mcg	OX-1 mcg	T-30 μg	CC-10 mcg
*Escherichia coli*	*R*	*S*	*S*	*R*	*S*	*S*	*R*	*S*	*R*	*R*
*Pseudomonas aeruginosa*	*S*	*R*	*R*	*S*	*R*	*S*	*S*	*S*	*R*	*S*
*Klebsiella pneumoniae*	*R*	*R*	*R*	*S*	*R*	*R*	*S*	*R*	*S*	*R*
*Staphylococcus aureus*	*S*	*R*	*R*	*R*	*S*	*S*	*R*	*S*	*R*	*S*
*Staphylococcus epidermidis*	*R*	*S*	*S*	*S*	*R*	*R*	*S*	*R*	*S*	*S*
*Bacillus cereus*	*R*	*R*	*S*	*R*	*R*	*R*	*R*	*S*	*S*	*R*
*Candida albicans*	*S*	*R*	*S*	*S*	*R*	*R*	*S*	*S*	*R*	*R*
*Candida tropicals*	*R*	*R*	*S*	*R*	*R*	*R*	*R*	*R*	*S*	*S*
*Candida glabrata*	*S*	*S*	*R*	*R*	*S*	*S*	*R*	*S*	*S*	*S*

a Resistant (*R*, clear zone ≤5 mm) or sensitive (*S*, inhibitory zones >5 mm).

#### 3.5.2. Antimicrobial activities survey

**Fig. 6:**
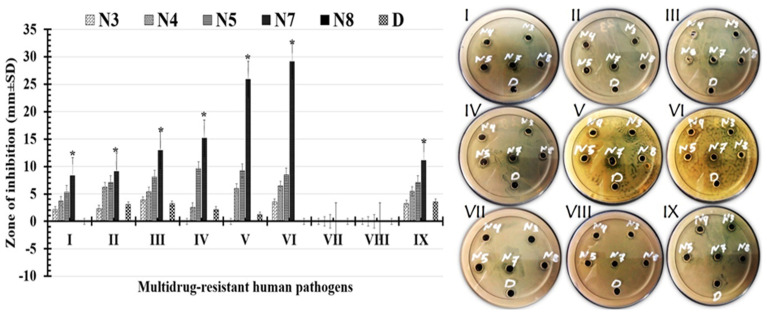
Images and charts for measured inhibition zones against (I): *Escherichia coli*, (II): *Pseudomonas aeruginosa*, (III): *Klebsiella pneumoniae*, (IV): *Staphylococcus aureus*, (V): *Staphylococcus epidermidis*, (VI): *Bacillus cereus*, (VII): *Candida albicans*, (VIII): *Candida tropicals*, and (IX) *Candida glabrata* recorded by (N3) for PCL/PVP/1.5% BT, (N4) for PCL/PVP/3% BT, (N5) for PCL/PVP/6% BT, (N7) for PCL/PVP/9% BT, (N8) for PCL/PVP and (D) for 1.5% BT. (*) Indicates the strongest inhibitory zones.

The results of the formulations' antimicrobial activity tests against nine multidrug-resistant human pathogens are shown in Fig. 6 and Table 3. The results indicated that, the tested formulations had a range of antimicrobial activities against all the pathogens tested, from weak to strong. Different pathogens responded differently to our formulations. The (PCL/PVP/9% BT) showed the widest zone of inhibition (potent activity), which was followed by the (PCL/PVP/6% BT) moderate activity. The formula (PCL/PVP/9% BT) under test was more efficient against Gram-positive bacteria than it is against Gram-negative bacteria. *Bacillus cereus* was the pathogen that the (PCL/PVP/9% BT) inhibited with the widest zone of inhibition (29.12 ± 3.65 mm), followed by *Staphylococcus epidermidis* (25.89 ± 4.21 mm). Additionally, the largest zone of inhibition for Gram-negative bacteria was exhibited by *Klebsiella pneumoniae* (12.96 ± 2.47 mm). Only (PCL/PVP/9% BT) formulation shows a high activity against *Candida glabrata* of (11.09 ± 4.64 mm), compared to other formulations. While, neither *Candida albicans* nor *Candida tropicalis* are inhibited by any of the tested formulations.

**Table 3:** Recorded inhibitory zones that produced by: (N3) for PCL/PVP/1.5% BT, (N4) for PCL/PVP/3% BT, (N5) for PCL/PVP/6% BT, (N7) for PCL/PVP/9% BT, (N8) for PCL/PVP and (D) for 1.5% BT against tested multidrug-resistant human pathogens^a^

Multidrug-resistant human pathogens	Zone of inhibition (mm ± SD)					
	N3	N4	N5	N7	N8	D
*Escherichia coli*	2.16 ± 0.54^bc^	3.69 ± 0.58^bc^	5.34 ± 0.97^ab^	8.31 ± 1.81^a^	0	0^bc^
*Pseudomonas aeruginosa*	2.34 ± 0.09^bc^	6.25 ± 0.48^bc^	7.06 ± 1.05^ab^	9.12 ± 2.35^a^	0	3.05 ± 1.54^bc^
*Klebsiella pneumoniae*	3.91 ± 0.11^bc^	5.37 ± 0.97^bc^	8.06 ± 2.25^ab^	12.96 ± 2.47^a^	0	3.21 ± 1.35^bc^
*Staphylococcus aureus*	0	2.56 ± 0.34^bc^	9.65 ± 1.36^ab^	15.17 ± 2.41^a^	0	2.14 ± 0.08^bc^
*Staphylococcus epidermidis*	0	5.97 ± 0.19^bc^	9.22 ± 1.89^ab^	25.89 ± 4.21^a^	0	1.2 ± 0.52^bc^
*Bacillus cereus*	3.54 ± 1.07^bc^	6.45 ± 0.68^bc^	8.47 ± 0.16^ab^	29.12 ± 3.65^a^	0	0
*Candida albicans*	0	0	0	0	0	0
*Candida tropicals*	0	0	0	0	0	0
*Candida glabrata*	3.25 ± 0.78^bc^	5.47 ± 1.02^bc^	7.12 ± 0.97^ab^	11.09 ± 4.64^a^	0	3.54 ± 1.06^bc^

a Different letters signify significant variations among treatments (*p* < 0.05).

#### 3.5.3. Anti-biofilm activities

In this study, both the microbial turbidity (OD_600_ nm ± SD) and viable cell count (log_10_ CFU ml^−1^ ± SD) were used to evaluate each formulation's ability (N3, N4, N5, and N7) to limit or prevent the growth of microbial pathogens. Significantly, N7-formula shows more powerful antimicrobial abilities than the N5-formula, maintaining the same results of the antimicrobial activities screened in the zone of inhibition test.

**Fig. 7:**
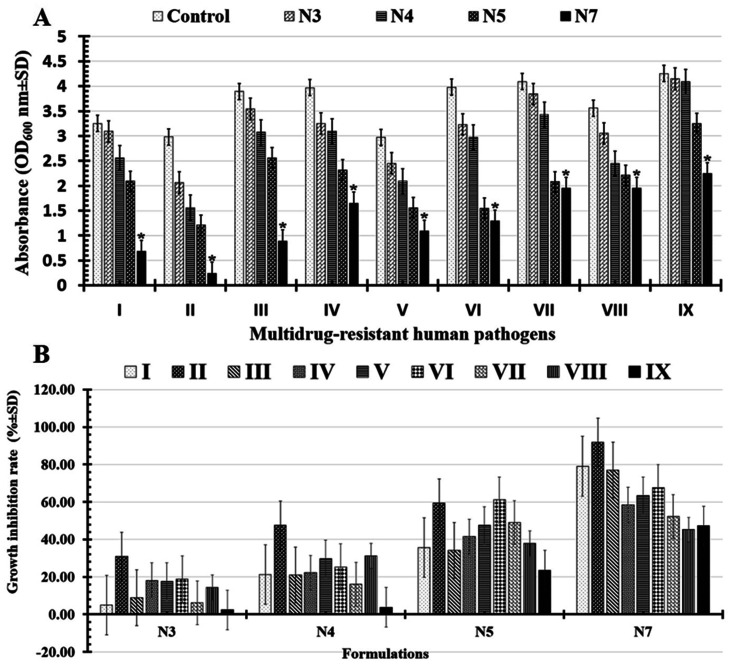
Illustrations charts for reported turgidities (A) as well as percentage of microbial growth reduction rates (B) of tested multi-drug human pathogens *Escherichia coli* (I), *Pseudomonas aeruginosa* (II), *Klebsiella pneumonia* (III), *Staphylococcus aureus* (IV), *Staphylococcus epidermidis* (V), *Bacillus cereus* (VI), *Candida albicans* (VII), *Candida tropical* (VIII), and *Candida glabrata* (IX) that treated with N3: PCL/PVP/1.5% BT, N4: PCL/PVP/3% BT, N5: PCL/PVP/6% BT, and N7: PCL/PVP/9% BT. (*) Indicates the most effective formula.

Significantly, it was determined that all the tested formulations' reported turgidities (OD_600_ nm) could prevent or slow the growth of biofilms in all the multi-drug-resistant human pathogens that had been studied (Fig. 7A and Table 4). As indicated in (Table 5 and Fig. 7B), the tested N7-formula shows a higher percentage of microbial growth reduction rate on Gram-negative bacteria such as *Pseudomonas aeruginosa* (91.94 ± 6.31), *Escherichia coli* (79.07 ± 6.92) and *Klebsiella pneumoniae* (77.12 ± 8.22). Additionally, *Bacillus cereus* showed the largest percentage of microbial growth reduction rates (67.58 ± 7.93%) among Gram-positive bacteria, followed by *Staphylococcus epidermidis* (63.29 ± 9.66%). Finally, the yeast cells under study had the lowest proportion of microbial growth decrease rates (Table 5 and Fig. 7B). These can be arranged in descending order of their percentages of microbial growth reduction rates as follows: *Candida albicans* (52.32 ± 2.74%), *Candida glabrata* (47.29 ± 4.12%), and *Candida tropicals* (45.22 ± 4.71).

**Table 4:** Recorded absorbance (OD_600_ nm ± SD) of the untreated (control) and the treated multidrug-resistant human pathogens with N3: PCL/PVP/1.5% BT, N4: PCL/PVP/3% BT, N5: PCL/PVP/6% BT, and N7: PCL/PVP/9% BT^a^

Multidrug-resistant human pathogens	Absorbance (OD_600_ nm ± SD)				
	Control	N3	N4	N5	N7
*Escherichia coli*	3.25 ± 0.45^a^	3.09 ± 0.56^a^	2.56 ± 0.35^ab^	2.09 ± 0.38^bc^	0.68 ± 0.62^c^
*Pseudomonas aeruginosa*	2.98 ± 0.56^a^	2.06 ± 0.23^a^	1.56 ± 0.64^ab^	1.21 ± 0.61^bc^	0.24 ± 0.61^c^
*Klebsiella pneumoniae*	3.89 ± 0.11^a^	3.54 ± 0.31^a^	3.07 ± 0.63^ab^	2.56 ± 0.42^bc^	0.89 ± 0.09^c^
*Staphylococcus aureus*	3.97 ± 1.52^a^	3.25 ± 0.14^a^	3.09 ± 0.23^ab^	2.32 ± 0.92^bc^	1.65 ± 0.02^c^
*Staphylococcus epidermidis*	2.97 ± 0.34^a^	2.45 ± 0.57^a^	2.09 ± 0.67^ab^	1.56 ± 0.65^bc^	1.09 ± 0.24^c^
*Bacillus cereus*	3.98 ± 0.03^a^	3.23 ± 0.64^a^	2.97 ± 0.97^ab^	1.55 ± 0.46^bc^	1.29 ± 0.25^c^
*Candida albicans*	4.09 ± 2.04^a^	3.84 ± 0.92^a^	3.43 ± 0.24^ab^	2.08 ± 0.41^bc^	1.95 ± 0.62^c^
*Candida tropicals*	3.56 ± 0.32^a^	3.05 ± 0.97^a^	2.45 ± 0.68^ab^	2.21 ± 0.32^bc^	1.95 ± 0.91^c^
*Candida glabrata*	4.25 ± 0.98^a^	4.15 ± 0.51^a^	4.09 ± 0.83^ab^	3.25 ± 0.36^bc^	2.24 ± 0.97^c^

a Significant differences between treatments are denoted by different letters (*p* < 0.05).

**Table 5:** Growth inhibition % rates for the multidrug-resistant human pathogens treated with N3: PCL/PVP/1.5% BT, N4: PCL/PVP/3% BT, N5: PCL/PVP/6% BT, and N7: PCL/PVP/9% BT^a^

Multidrug-resistant human pathogens	Growth inhibition rate (% ± SD)			
	N3	N4	N5	N7
*Escherichia coli*	4.92 ± 3.07^c^	21.23 ± 7.69^c^	35.69 ± 2.31^b^	79.07 ± 6.92^a^
*Pseudomonas aeruginosa*	30.87 ± 2.48^c^	47.65 ± 1.67^c^	59.39 ± 5.97^b^	91.94 ± 6.31^a^
*Klebsiella pneumoniae*	8.99 ± 0.74^c^	21.07 ± 9.69^c^	34.19 ± 2.31^b^	77.12 ± 8.22^a^
*Staphylococcus aureus*	18.13 ± 6.02^c^	22.16 ± 6.24^c^	41.56 ± 1.71^b^	58.43 ± 8.28^a^
*Staphylococcus epidermidis*	17.50 ± 8.41^c^	29.62 ± 9.62^c^	47.45 ± 11.75^b^	63.29 ± 9.66^a^
*Bacillus cereus*	18.84 ± 4.22^c^	25.37 ± 6.88^c^	61.05 ± 5.27^b^	67.58 ± 7.93^a^
*Candida albicans*	6.11 ± 2.46^c^	16.13 ± 6.91^c^	49.14 ± 4.25^b^	52.32 ± 2.74^a^
*Candida tropicals*	14.32 ± 5.84^c^	31.17 ± 9.77^c^	37.92 ± 1.34^b^	45.22 ± 4.71^a^
*Candida glabrata*	2.35 ± 2.94^c^	3.76 ± 4.70^c^	23.52 ± 9.41^b^	47.29 ± 4.12^a^

a Different letters (*p* < 0.05) are used to indicate significant differences between treatments.

**Fig. 8:**
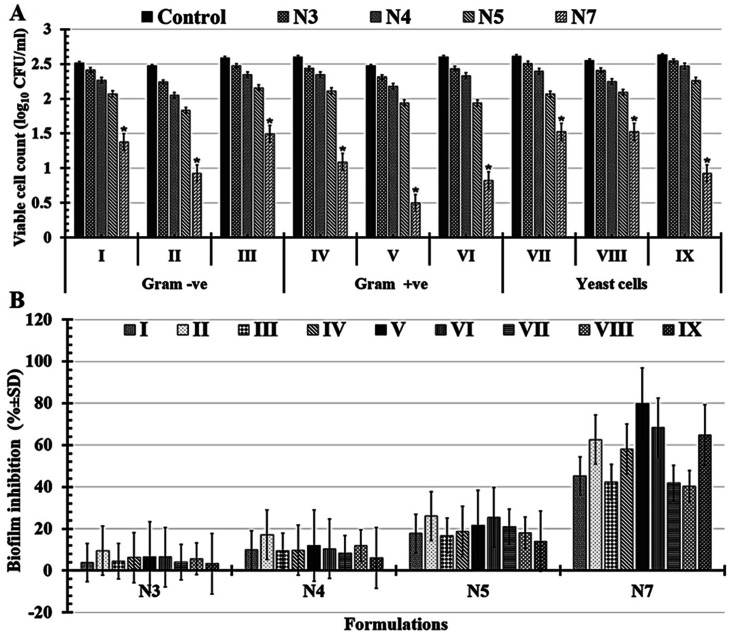
Charts of reported viable cell count (A) as well as the percentage of biofilm inhibition rates (B) of tested multi-drug human pathogens *e.g. Escherichia coli* (I), *Pseudomonas aeruginosa* (II), *Klebsiella pneumonia* (III), *Staphylococcus aureus* (IV), *Staphylococcus epidermidis* (V), *Bacillus cereus* (VI), *Candida albicans* (VII), *Candida tropical* (VIII), and *Candida glabrata* (IX) that untreated (control) and treated with N3: PCL/PVP/1.5% BT, N4: PCL/PVP/3% BT, N5: PCL/PVP/6% BT, and N7: PCL/PVP/9% BT. (*) designates the most efficient formula.

Significantly, it was shown that all tested formulations (N3, N4, N5, and N7) could reduce the severity of biofilms in all tested multi-drug-resistant human pathogens compared with D-treatment (1.5% BT) based on the reported viable cell counts Fig. 8A and percentages of biofilm inhibition rates in Fig. 8B. Notably, N7-formula still outperforms the N5-formula in antimicrobial activity against all of the tested multi-drug-human pathogens. It's interesting to note that N7-formula is semi-toxic to yeast cells and Gram-negative bacteria but extremely toxic to Gram-positive bacteria when evaluated by the number of viable colonies Table 6 and Fig. 8A. Among Gram-positive bacteria, *Staphylococcus epidermidis* had the lowest viable cell count, going to drop from 2.47 ± 0.27 to 0.49 ± 0.08 CFU ml^−1^ after being treated with N7-formula. When compared to other Gram-positive bacteria, *Staphylococcus epidermidis* and *Bacillus cereus* both had high levels of biofilm inhibition (79.84 ± 7.97% and 68.35 ± 15.90%; respectively), as shown in Table 7 and Fig. 8B. *Candida glabrata* (64.83 ± 4.75%) and *Pseudomonas aeruginosa* (62.64 ± 3.55%) both had nearly equivalent moderate levels of biofilm inhibition among the tested yeast cells and Gram-negative bacteria, as shown in Table 7 and Fig. 8B. Thus, these four human pathogens (*Staphylococcus epidermidis*, *Bacillus cereus*, *Candida glabrata*, and *Pseudomonas aeruginosa*) were extensively examined using N7-formula to determine the time-kill kinetics in the following section.

**Table 6:** Viable cell count for the multidrug-resistant human pathogens that treated with N3: PCL/PVP/1.5% BT, N4: PCL/PVP/3% BT, N5: PCL/PVP/6% BT, and N7: PCL/PVP/9% BT^a^

Multidrug-resistant human pathogens		Viable cell count (log_10_ CFU ml^−1^)				
		Control	N3	N4	N5	N7
Gram -ve	*Escherichia coli*	2.51 ± 1.88^a^	2.41 ± 0.42^ab^	2.26 ± 0.55^bc^	2.06 ± 0.83^c^	1.37 ± 0.65^d^
	*Pseudomonas aeruginosa*	2.47 ± 0.42^a^	2.23 ± 0.85^ab^	2.05 ± 0.45^bc^	1.83 ± 0.97^c^	0.92 ± 0.04^d^
	*Klebsiella pneumoniae*	2.58 ± 0.99^a^	2.47 ± 0.28^ab^	2.34 ± 0.44^bc^	2.15 ± 0.64^c^	1.49 ± 0.34^d^
Gram +ve	*Staphylococcus aureus*	2.59 ± 0.87^a^	2.43 ± 0.61^ab^	2.34 ± 0.72^bc^	2.11 ± 0.36^c^	1.08 ± 0.81^d^
	*Staphylococcus epidermidis*	2.47 ± 0.27^a^	2.31 ± 0.34^ab^	2.17 ± 0.74^bc^	1.94 ± 0.13^c^	0.49 ± 0.08^d^
	*Bacillus cereus*	2.59 ± 0.98^a^	2.43 ± 0.34^ab^	2.33 ± 0.08^bc^	1.93 ± 0.08^c^	0.82 ± 0.28^d^
Yeast cells	*Candida albicans*	2.61 ± 0.17^a^	2.50 ± 0.86^ab^	2.39 ± 0.26^bc^	2.06 ± 0.62^c^	1.52 ± 0.17^d^
	*Candida tropicals*	2.55 ± 0.14^a^	2.40 ± 0.85^ab^	2.24 ± 0.64^bc^	2.09 ± 0.25^c^	1.52 ± 0.17^d^
	*Candida glabrata*	2.62 ± 0.83^a^	2.54 ± 0.23^ab^	2.46 ± 0.90^bc^	2.26 ± 0.07^c^	0.92 ± 0.42^d^

a Different letters (*p* < 0.05) are used to indicate significant differences between treatments.

**Table 7:** Biofilm inhibition % (% ± SD) for the multidrug-resistant human pathogens that treated with N3: PCL/PVP/1.5% BT, N4: PCL/PVP/3% BT, N5: PCL/PVP/6% BT, and N7: PCL/PVP/9% BT^a^

Multidrug-resistant human pathogens	Biofilm inhibition (% ± SD)			
	N3	N4	N5	N7
*Escherichia coli*	3.88 ± 7.34^c^	9.80 ± 5.82^bc^	17.65 ± 8.02^b^	45.19 ± 7.41^a^
*Pseudomonas aeruginosa*	9.54 ± 1.19^c^	17.12 ± 7.05^bc^	25.99 ± 7.84^b^	62.64 ± 3.55^a^
*Klebsiella pneumoniae*	4.50 ± 4.67^c^	9.47 ± 8.12^bc^	16.73 ± 8.61^b^	42.33 ± 6.40^a^
*Staphylococcus aureus*	6.25 ± 7.82^c^	9.67 ± 3.56^bc^	18.66 ± 9.33^b^	58.12 ± 9.13^a^
*Staphylococcus epidermidis*	6.44 ± 2.65^c^	11.94 ± 1.23^bc^	21.49 ± 1.95^b^	79.84 ± 7.97^a^
*Bacillus cereus*	6.43 ± 0.64^c^	10.37 ± 0.71^bc^	25.43 ± 8.19^b^	68.35 ± 15.90^a^
*Candida albicans*	3.94 ± 0.80^c^	8.38 ± 0.89^bc^	20.88 ± 5.51^b^	41.73 ± 2.27^a^
*Candida tropicals*	5.59 ± 0.95^c^	11.95 ± 2.82^bc^	17.98 ± 4.63^b^	40.35 ± 5.81^a^
*Candida glabrata*	3.27 ± 0.43^c^	6.06 ± 2.71^bc^	14.01 ± 3.53^b^	64.83 ± 4.75^a^

a To indicate significant differences between treatments, different letters (*p* < 0.05) were used.

#### 3.5.4. Evaluation of time-kill kinetics

**Fig. 9:**
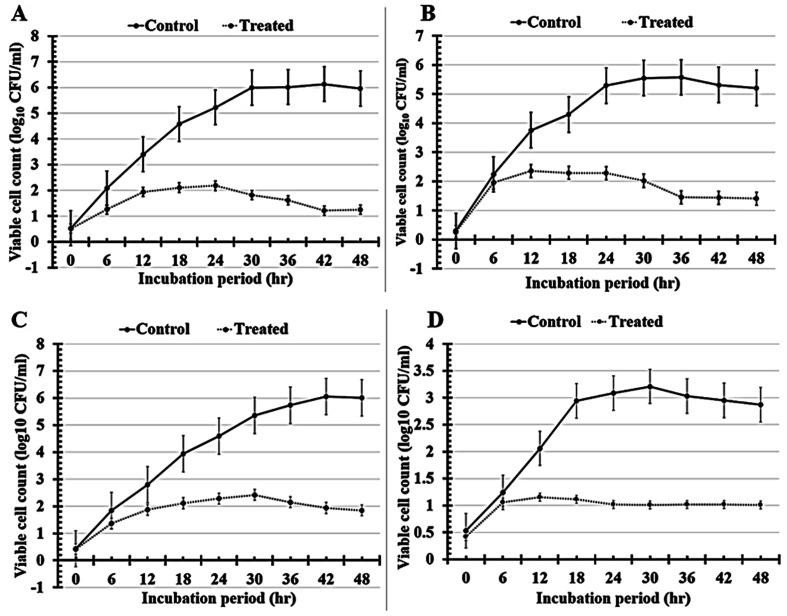
The growth patterns of the untreated (control) and treated multi-drug resistant human pathogens *Staphylococcus epidermidis* (A), *Bacillus cereus* (B), *Candida glabrata* (C), and *Pseudomonas aeruginosa* (D) with N7 (PCL/PVP/9% BT) which was used to calculate time-kill kinetics.

In the current study, time-kill analyses were used to compare the killing rates of the tested human pathogens that treated with N7-formula to untreated cells (control), as shown by the curves in Fig. 9 and the tabulated results in Table 8. The maximum growth reduction value for *Staphylococcus epidermidis* treated with N7-formula was found to be 80.26 ± 1.11% after 42 incubation hours Fig. 9A and Table 8. Additionally, the highest percentage of growth reduction for *Bacillus cereus* was observed to be 73.98 ± 2.35% after 36 h of incubation periods Fig. 9B. After 48 h of incubation, the best growth reduction value for *Candida glabrata* was found to be 69.21 ± 7.07% Fig. 9C, followed by *Pseudomonas aeruginosa* (68.54 ± 1.32) which was recorded after 30 h of N7-formula treatment Fig. 9D and Table 8. Finally, each of our tested antimicrobial analyses proves that applying N7-formula effectively inhibits the growth of multi-drug resistant human pathogens.

**Table 8:** The results of the time-kill kinetics of the tested multidrug-resistant human pathogens (*Staphylococcus epidermidis*, *Bacillus cereus*, *Candida glabrata*, and *Pseudomonas aeruginosa*) that treated with N7 (PCL/PVP/9% BT) that was used to evaluate the time-kill kinetics. Every data point is a mean ± standard deviation of the mean that was determined using three different tests

Cultivation period (h)	Multidrug-resistant human pathogens treated with N7-formula			
	Viable cell count (log_10_ CFU ml^−1^ ± SD)	Growth reduction (% ± SD)	Viable cell count (log_10_ CFU ml^−1^ ± SD)	Growth reduction (% ± SD)
	***Staphylococcus epidermidis***		***Bacillus cereus***	
0	0.52 ± 0.01	1.88 ± 0.67	0.25 ± 0.09	13.66 ± 6.67
6	1.26 ± 0.09	39.51 ± 14.65	1.96 ± 0.12	12.61 ± 8.51
12	1.94 ± 0.51	42.93 ± 5.61	2.35 ± 1.42	37.38 ± 8.08
18	2.11 ± 0.22	53.90 ± 6.67	2.29 ± 1.08	46.67 ± 2.26
24	2.18 ± 1.42	58.27 ± 12.36	2.28 ± 0.82	56.85 ± 3.88
30	1.82 ± 0.07	69.61 ± 8.82	2.02 ± 1.05	63.60 ± 7.21
36	1.62 ± 0.14	73.05 ± 9.45	1.45 ± 0.014	73.98 ± 2.35
42	1.21 ± 0.09	80.26 ± 1.11	1.44 ± 0.054	72.88 ± 1.35
48	1.25 ± 0.87	79.02 ± 6.84	1.41 ± 0.58	72.93 ± 6.26

	***Candida glabrata***		***Pseudomonas aeruginosa***	
0	0.42 ± 0.01	2.32 ± 0.55	0.42 ± 0.12	20.75 ± 4.71
6	1.36 ± 0.95	26.20 ± 8.79	1.06 ± 0.32	14.72 ± 5.23
12	1.87 ± 0.97	33.13 ± 4.96	1.15 ± 0.92	44.06 ± 8.65
18	2.11 ± 0.52	46.42 ± 3.24	1.11 ± 0.65	62.17 ± 1.69
24	2.28 ± 1.02	50.27 ± 2.05	1.02 ± 0.94	66.97 ± 9.65
30	2.42 ± 0.68	54.77 ± 10.13	1.01 ± 0.97	68.54 ± 1.32
36	2.15 ± 0.52	62.48 ± 7.45	1.01 ± 0.51	66.51 ± 7.21
42	1.94 ± 0.35	67.93 ± 3.84	1.02 ± 0.65	65.42 ± 3.72
48	1.85 ± 1.02	69.21 ± 7.97	1.01 ± 0.02	64.88 ± 3.62

## 4 Conclusions

The outcomes of this work could support the assumption that using BT in a newly repurposed use as an antimicrobial agent might be auspicious. Herein, BT-loaded PCL/PVP NFs were successfully prepared with different drug concentrations *via* electrospinning technique. The BT-PCL/PVP NFs showed smooth surfaces and the one prepared with 9% BT which have the smallest diameter of about 80 nm, compared to other nanofibers. All scaffolds' release profiles confirmed their prolonged manners that lasted for 7 days. They also demonstrated satisfactory antimicrobial activities against several human pathogens. Based on the viable cell counts results, it was observed that all the fabricated BT-loaded scaffolds could reduce the biofilm formation in all tested multi-drug-resistant human pathogens when compared to the free BT (1.5%). However, the BT-loaded nanofibers fabricated with 9% BT showed the maximum growth reduction value of 80% for *Staphylococcus epidermidis* after 42 h, 74% for *Bacillus cereus* after 36 h, 69% for *Candida glabrata* after 48 h, and 68% for *Pseudomonas aeruginosa* after 30 h. In conclusion, BT-loaded PCL/PVP NFs and particularly 9% BT loaded PCL/PVP NFs one could be as an efficient biomaterials candidate for antimicrobial applications which would endow eligibility for upcoming pre-clinical and clinical researches.

## Data availability

The datasets generated or analyzed during the current study are available from the corresponding author upon reasonable request.

## Author contributions

All authors contributed to the study's conception and design. Material preparation, data collection, and analysis were performed by all authors. All authors participated in the writing of the manuscript. All authors read and approved the final manuscript.

## Conflicts of interest

All authors declare they have no financial or non-financial interests. The authors have no relevant financial or non-financial interests to disclose.

## Supplementary Material
